# Superior Sleeve Avulsion of the Patella

**DOI:** 10.5334/jbsr.1597

**Published:** 2018-10-05

**Authors:** Wouter Hemeleers, Wim Siemons

**Affiliations:** 1 Ziekenhuis Oost-Limburg, BE

**Keywords:** sleeve avulsion, patella

A 19-year-old man visited the emergency room with persistent pain in the left knee after falling a few weeks earlier.

Clinical examination showed that flexion of the knee was possible, up to 50 degrees. The tendon of the quadriceps muscle appeared pressure sensitive while the upper pole of the patella was painless.

A standard X-ray (Figure 1, lateral view) and an ultrasound (Figure 2), of the knee were requested, and showed a thin, crescent-shaped bony fragment at the superior aspect of the patella (arrows), consistent with a sleeve avulsion fracture. The patient was treated conservatively with NSAIDs, application of ice and temporary suspension of sporting activities.

**Figure 1:**
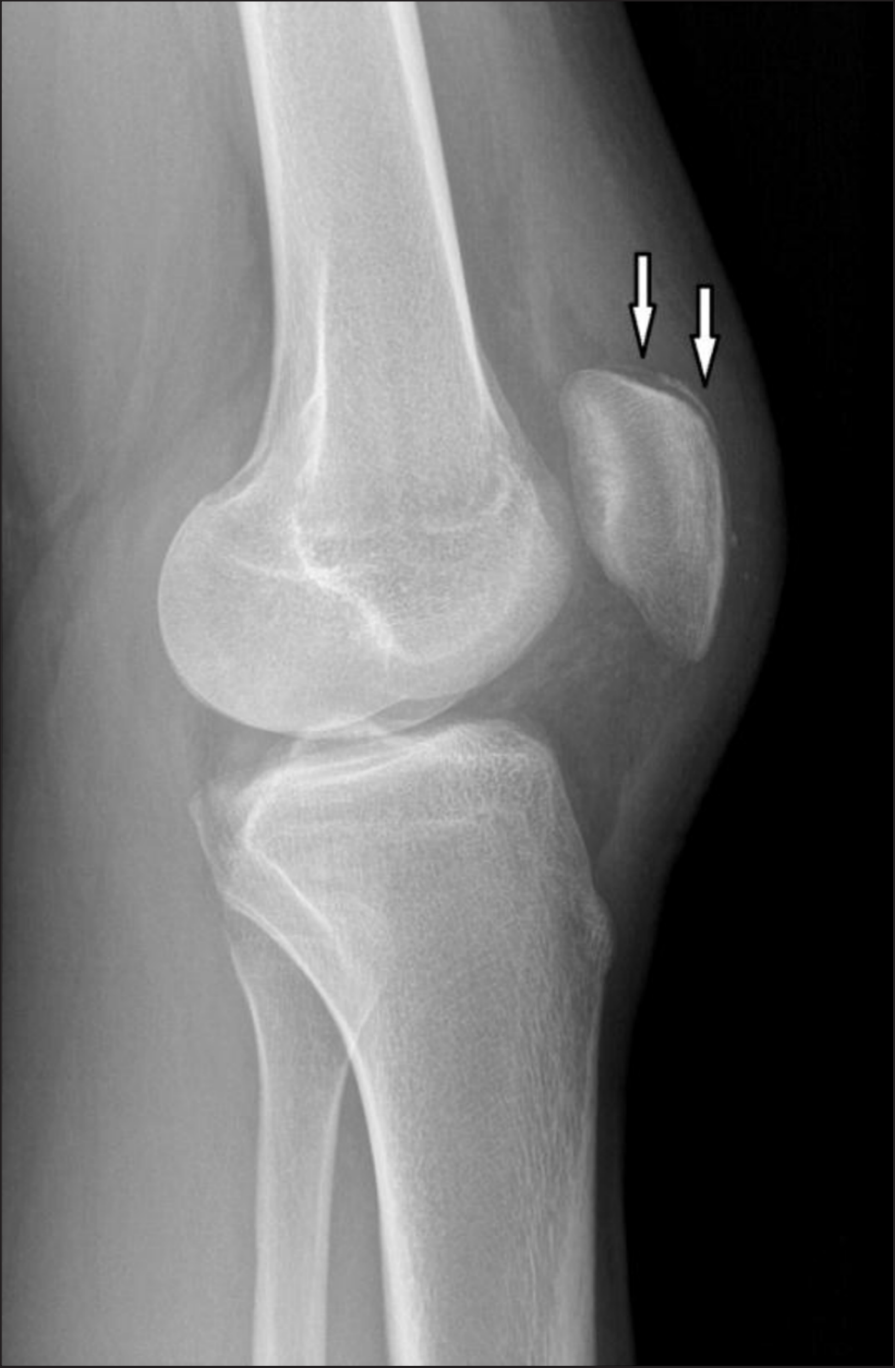
Lateral X-ray of the knee shows a linear bony fragment at the upper pole of the patella.

**Figure 2:**
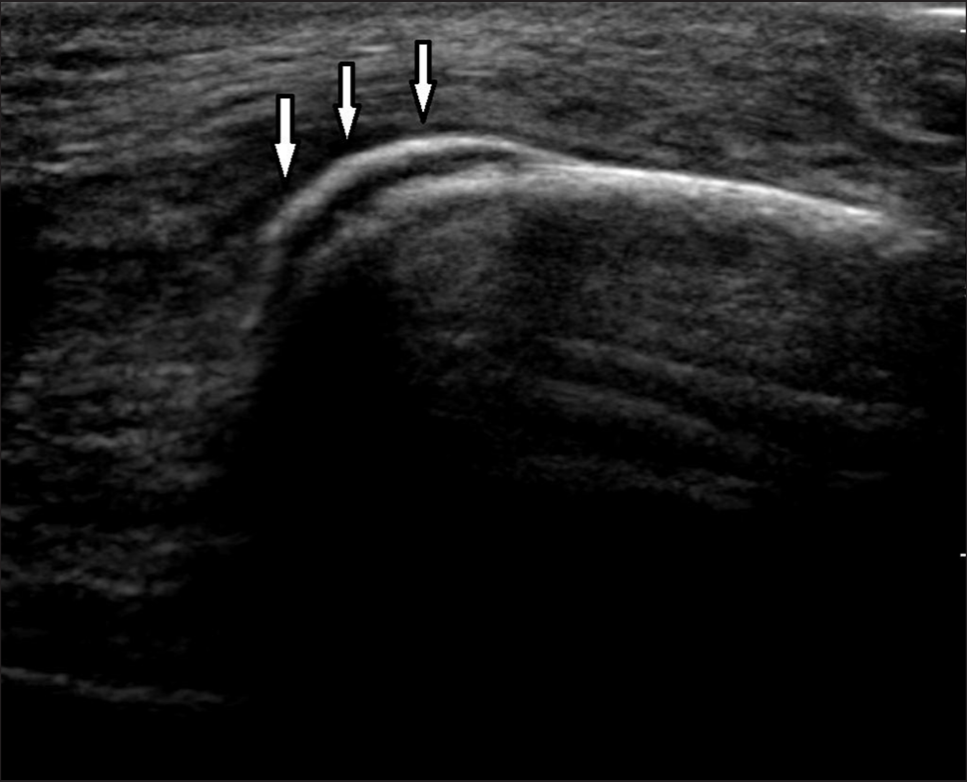
Ultrasound of the suprapatellar region shows a crescent-shaped echogenic fragment at the upper pole of the patella.

## Comment

Sleeve avulsion fractures of the patella mostly occur in children and adolescents because of their skeletal immaturity. Patellar fractures account for 1% of all pediatric fractures. They are extremely rare in patients with a completely matured skeleton. Sleeve avulsion fractures of the upper pole are much less frequent in comparison to the lower pole. Adolescents are the most susceptible because of their rapid growth and participation in high-activity sports. Hyperextension trauma or a direct hit on the knee are the most common causes [1].

Knee radiographs can show a small bony fragment at the superior pole of the patella. However, since the avulsed fragment mostly consists of cartilage and sometimes periosteal tissue, it can be easily missed, with the only potential findings including soft-tissue swelling, joint effusion and *patella baja*. Late radiographs can still prove an avulsion fracture after ossification of the avulsed periosteal tissue.

Ultrasound is a valuable alternative if standard X-ray images of the knee prove to be negative. It has excellent detection of cartilaginous lesions and adjacent soft-tissue swelling. MRI is excellently suited for evaluation of this kind of fracture. MRI can help to determine the size of the avulsed fragment in addition to assessing the size of the chondral fragment, integrity of the articular surface of the patella, the extent of periosteal avulsion, and the relationship of the fracture fragments.

Conservative treatment should only be considered in fractures in which the displacement is not greater than 2mm, as the results of conservative treatment remain unsatisfactory for larger gaps.
